# What if cancer survival in Britain were the same as in Europe: how many deaths are avoidable?

**DOI:** 10.1038/sj.bjc.6605401

**Published:** 2009-12-03

**Authors:** M Abdel-Rahman, D Stockton, B Rachet, T Hakulinen, M P Coleman

**Affiliations:** 1 Cancer Research UK Cancer Survival Group, Non-Communicable Disease Epidemiology Unit, Department of Epidemiology and Population Health, London School of Hygiene and Tropical Medicine, Keppel Street, London WC1E 7HT, UK; 2 Faculty of Mathematical Sciences, University of Khartoum, Khartoum, Sudan; 3 Scottish Public Health Observatory, Information Services Division (ISD) Scotland, Gyle Square, 1 South Gyle Crescent, Edinburgh, Scotland, UK; 4 Finnish Cancer Registry, Pieni Roobertinkatu 9, Helsinki, Finland

**Keywords:** Britain, Europe, survival, avoidable mortality, health policy

## Abstract

**Objective:**

To estimate the number of deaths among cancer patients diagnosed in Great Britain that would be avoidable within 5 years of diagnosis if the mean (or highest) survival in Europe for patients diagnosed during 1985–1989, 1990–1994 and 1995–1999 were achieved.

**Design:**

Five-year relative survival for cancers in Great Britain compared with that from other countries in the EUROCARE-2, -3 and -4 studies. Calculation of excess deaths (those more than expected from mortality in the general population) that would be avoidable among cancer patients in Britain if relative survival were the same as in Europe.

**Setting:**

Great Britain (England, Wales, Scotland) and 13 other European countries.

**Subjects:**

2.8 million adults diagnosed in Britain with 1 of 39 cancers during 1985–1989 (followed up to 1994), 1990–1994 (followed up to 1999) and 1995–1999 (followed up to 2003).

**Main outcome measure:**

Annual number of avoidable deaths within 5 years of diagnosis. Percentage of the excess (cancer-related) deaths among cancer patients that would be avoidable.

**Results:**

Compared with the mean European 5-year relative survival, the largest numbers of avoidable deaths for patients diagnosed during 1985–1989 were for cancers of the breast (about 18% of the excess mortality from this cancer, 7541 deaths), prostate (14%, 4285), colon (9%, 4090), stomach (8%, 3483) and lung (2%, 3548). For 1990–1994, the largest numbers of avoidable deaths were for cancers of the prostate (20%, 7335), breast (15%, 6165), colon (9%, 4376), stomach (9%, 3672), lung (2%, 3735) and kidney (22%, 2644). For 1995–1999, most of the avoidable deaths were for cancers of the prostate (17%, 5758), breast (15%, 5475), lung (3%, 4923), colon (10%, 4295), stomach (9%, 3137) and kidney (21%, 2686).

Overall, some 6600–7500 premature deaths would have been avoided each year among cancer patients diagnosed in Britain during 1985–1999 if the mean survival in Europe had been achieved. This represents 6–7% of cancer-related mortality. Compared with the highest European survival, avoidable premature mortality among cancer patients fell from about 12 800 deaths a year (12.2% of cancer-related mortality) to about 11 400 deaths a year (10.6%) over the same period.

A large component of the avoidable mortality is due to prostate cancer: excluding this cancer from comparison with the European mean survival reduces the annual number of avoidable deaths by 1000–1500, and the percentage of excess mortality by up to 1%. Compared with the highest survival, the annual number of avoidable deaths would be 1500–2000 fewer, and 1–2% lower as a percentage of excess mortality, but the overall trend in avoidable premature mortality among cancer patients would be similar, falling from 11.4% (1985–1989) to 10.3% (1990–1994) and 9.7% for those diagnosed during 1995–1999.

For several cancers, survival in Britain was slightly higher than the mean survival in Europe; this represented some 110–180 premature deaths avoided each year during the period 1985–2003.

**Conclusions:**

Avoidable premature mortality among cancer patients diagnosed in Britain during 1985–1999 has represented 6–7% of cancer-related mortality compared with the mean survival in Europe. Compared with the highest levels of survival in Europe, the reduction from 12.2% to 10.6% of cancer-related mortality reflects small but steady progress over the period 1985–2003.

The EUROCARE study has provided population-based survival estimates for up to 20 European countries for adults (15–99 years) diagnosed with cancer since 1978 (Berrino *et al*, 1995b, 1999b, 2003, 2007). It has shown continuing increases in survival but large and persistent international variations across Europe. Data from England and Scotland have been included in all the EUROCARE studies, data from Wales for patients diagnosed since 1990, and from Northern Ireland since 1995. Survival for most adult cancers in the United Kingdom has generally been lower than in comparable western European countries.

Concern about the cancer survival deficit in Britain (Department of Health, 1999) contributed to development of the NHS Cancer Plan (Department of Health, 2000), which envisaged a 20% reduction in cancer death rates under age 75 by 2010. It has been suggested that 10 000 lives a year would have been saved if 5-year survival for patients diagnosed in Britain during 1985–1989 had reached the European average, and 25 000 lives a year if survival were as high as the best in Europe, but no details were given (Sikora, 1999). The question of how many cancer deaths would be avoidable if survival in Britain were at the level seen in other EU countries has been raised in Parliament. It could not be answered (Kelly, 2002).

The EUROCARE-4 study provided relative survival estimates from 20 European countries for adults diagnosed with 1 of 39 different malignancies during 1995–1999 and followed up to 2001. This enables assessment of trends in avoidable cancer mortality in Britain over the period 1985–1999, based on data from the EUROCARE-2, -3 and -4 studies.

We set out to estimate how many cancer deaths would have been avoided within 5 years of diagnosis if survival among patients diagnosed in Great Britain (England, Scotland and Wales) during 1985–1989, 1990–1994 and 1995–1999 had been equivalent either to the mean survival or to the highest survival seen in other European countries. Cancer survival has been improving in most European countries, so comparison with a shifting baseline is appropriate. Trends in avoidable mortality can be seen as an overall comparative measure of progress in cancer control between Britain and the rest of Europe.

## Materials and methods

Detailed cancer survival data from the EUROCARE-2, -3 and -4 studies are available by sex and age at diagnosis (15–44, 45–54, 55–64, 65–74, 75–99 years) (Berrino *et al*, 1999a, 2007; Carrani *et al*, 1999; Roazzi *et al*, 2003). The cancers included represent about 93% of all malignant neoplasms (excluding non-melanoma skin cancer) diagnosed in adults in Britain during 1985–1999; the remaining 7% are mostly ill defined, and avoidable deaths from these cancers were not estimated.

Thirteen of the 19 European countries outside the United Kingdom that participated in EUROCARE-4 also participated in the EUROCARE-2 and EUROCARE-3 studies. We used data from these 13 countries to simplify the interpretation of changes in avoidable mortality. Population coverage by contributing registries was unchanged in four countries with national coverage, but it changed (usually increased) between successive EUROCARE studies in the other nine countries (Table 1). We used all the data from these 13 countries, rather than restrict the comparison to the individual cancer registries that contributed to all three studies.

The European mean 5-year survival was calculated for each cancer, age group and sex, as the mean of the survival estimates from the 13 countries, weighted by the proportion of patients included in the EUROCARE-4 data for that country, for that cancer and that sex. This simply reflects the different size of the data sets from each country: it does not assume that regional survival figures can be extrapolated nationally. Using a fixed set of weights avoids the artificial change in the European mean survival that would otherwise occur as a result of the varying proportional contributions by different countries to successive EUROCARE studies. To avoid bias, data from England, Scotland and Wales were not used in calculating the European mean survival, because they constituted a large fraction of the total data for Europe. For the less common cancers in some of the smaller data sets, age–sex cells occasionally contained small numbers of patients: if the 5-year survival estimate in such cells was zero or missing, we substituted the survival estimate for the adjacent age group for that country, cancer and sex.

The ‘highest’ European survival in each calendar period was identified as follows. First, as with the European mean survival, we excluded survival in England, Scotland and Wales. Second, to avoid criticism levelled at the data from Austria and Switzerland, where survival has tended to be high, we also excluded data from those countries. Further, to avoid giving undue emphasis to extremely high survival observed in any one region or country among the remaining 11 countries, we identified, for each cancer, the three countries with the highest age–sex-standardised relative survival. The frequency with which countries contributed to the highest European survival estimate under these constraints is shown in Table 1. For a given cancer, age group and sex, the highest European survival was then taken as the average of the survival estimates in those three countries, weighted by their contribution to the EUROCARE-4 data. If a component estimate was not available, the same tactic was used as for the mean European survival.

National data (100% coverage) were available from Scotland for all three studies. In England, 7 of the then 11 English regional cancer registries contributed data on adults diagnosed during 1985–1989 to the EUROCARE-2 study (49.6% population coverage). Survival in the English regions that contributed to EUROCARE-2 was generally similar to that in England and Wales as a whole. Coverage of England rose to 62.6% for EUROCARE-3 (1990–1994) and to 100% for EUROCARE-4 (1995–1999).

Data for Wales were not included in EUROCARE-2, but national data (100% coverage) were included in EUROCARE-3 and -4. The population of Wales (2.9 million) is about 6% that of England, and differences in survival between Wales and England for most cancers were not large, either for patients diagnosed during the period 1980–1990 (Coleman *et al*, 1999), or for those diagnosed during 1990–1994 (Sant *et al*, 2003). To estimate avoidable cancer deaths during 1985–1989 for Great Britain as a whole, including Wales, we assumed that cancer incidence and survival in Wales in that period had been the same as in England for each cancer, sex and age group. In effect, the number of avoidable deaths estimated for England for the period 1985–1989 was inflated by some 6% to account for the population of Wales. We checked the impact of this approach by using it for 1990–1994 (EUROCARE-3) and 1995–1999 (EUROCARE-4): it gave very similar results to those obtained from the data that were actually contributed by Wales to those studies (results not shown).

**Figure 1:**
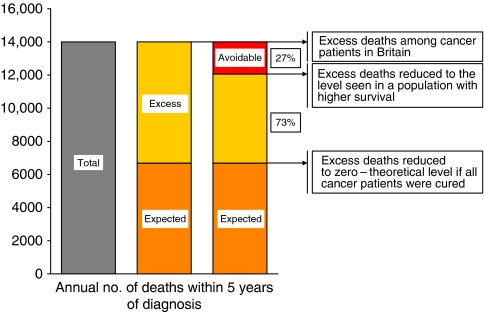
Partition of the annual number of deaths in cancer patients within 5 years of diagnosis into the number expected from background mortality and the excess deaths (attributable to cancer), showing the proportion of the excess deaths that would be avoidable (27%) if relative survival had reached the higher level seen in a comparator population. Note: numbers are of deaths occurring in cancer patients, not deaths certified as due to the cancer in question (see text).

The overall mortality in a cohort of cancer patients can be divided into two components, the background mortality (expected from all-cause death rates in the general population), and the excess mortality, which is then attributable to the cancer (Figure 1). Relative survival reflects the excess mortality among cancer patients, over and above the background mortality in the country or region where they live (Berkson and Gage, 1950; Estève *et al*, 1990, 1994); background mortality varies two-fold or more across Europe (Micheli *et al*, 1999). ‘Avoidable’ deaths are then the component of excess (cancer-related) mortality that would not occur if relative survival were at the higher level seen in a comparator population, instead of what was actually observed. In Figure 1, for example, avoidable deaths comprise 27% of the overall excess mortality.

The number of avoidable deaths was calculated separately for England, Scotland and Wales, for each age group and sex, and for each cancer, against both the mean and the highest survival in Europe. Avoidable deaths within 5 years of diagnosis are expressed both as the absolute number of deaths per year and as the percentage of the excess mortality for each cancer and calendar period. We refer to this avoidable mortality within 5 years of diagnosis as the number or proportion of ‘avoidable premature deaths’ in cancer patients.

A standardisation approach was used (Richards *et al*, 2000). Briefly, for each cancer, sex and age group, the number of avoidable deaths within 5 years of diagnosis was calculated as the difference in 5-year relative survival between the value for England, or Scotland, or Wales, and the corresponding aggregate (mean or highest) value for Europe, multiplied by the expected survival and the total number of incident cases in England, Scotland or Wales for that age group and sex (see Appendix). As cancer patients may die of causes other than cancer, the number of avoidable deaths is calculated by applying the difference in relative survival only to the expected number of survivors based on the background mortality, and not to the total number of patients. The avoidable deaths for each cancer, age group, sex and country were then summed to produce the total for Britain.

It has been argued that the EUROCARE study produces under-estimates of the true level of survival in Britain (or conversely that survival in other countries is too high). We therefore carried out some sensitivity analyses, by re-computing the avoidable mortality after assuming that 5-year relative survival in England, Scotland and Wales was either 2% or 3% higher, for each cancer and for each sex and age group, than was actually reported in the EUROCARE studies.

Results are presented separately for 22 common malignancies, including the four main types of leukaemia combined (Table 2). Results for 17 less common cancers examined in the EUROCARE study were calculated separately, but are presented as a combined group (‘other cancers’).

## Results

### European mean survival

**Figure 2:**
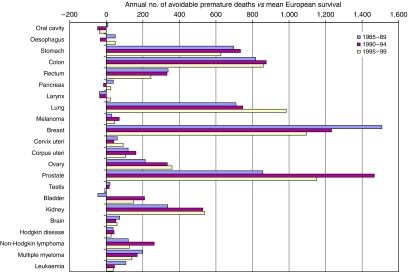
Annual number of deaths within 5 years of diagnosis that would be avoidable among cancer patients in Britain if relative survival were equal to the mean European survival: 22 common cancers, patients diagnosed 1985–1989, 1990–1994, 1995–1999,

Among the 839 551 adults (15–99 years) diagnosed with one of the cancers included in the study in Great Britain during the 5 years 1985–1989, there were 526 270 deaths in excess of the background mortality in the general population within 5 years of diagnosis. For those cancers with lower 5-year survival in Britain than in Europe, 33 071 of these excess deaths would have been avoided if European mean survival had been achieved for each cancer in each sex and age group (Table 2; Figure 2). This represents 6614 premature cancer deaths per year, or 6.3% of the overall excess mortality for these cancers. For those cancers for which 5-year survival was generally higher in Britain, 568 premature deaths were avoided, or 114 deaths per year.

Among the 966 518 cancer patients diagnosed during 1990–1994, there were 560 718 excess deaths within 5 years of diagnosis. For the cancers with lower survival in Britain, 37 620 of these excess deaths would have been avoided if the mean 5-year relative survival observed in the 13 other European countries had been achieved. This represents 7524 premature deaths per year, or 6.7% of the excess mortality in cancer patients within 5 years. Where survival in Britain was generally higher than in Europe, 903 premature deaths were avoided, or 181 per year.

Of the 1 020 786 cancer patients diagnosed during 1995–1999, 539 934 excess deaths occurred within 5 years of diagnosis. For cancers with lower survival in Britain, about 34 841 of these excess deaths would have been avoided if the mean European survival had been achieved. This represents 6968 premature deaths a year, or 6.5% of the overall excess mortality from these cancers. Among the several cancers for which survival in Britain was generally higher than in Europe, 850 premature deaths were avoided, or 170 per year.

For patients diagnosed during 1985–1989, the largest numbers of avoidable deaths within 5 years of diagnosis arose for cancers of the breast (7541, about 18% of excess deaths from this cancer), prostate (4285, 14%), colon (4090, 9%), lung (3548, 2%) and stomach (3483, 8%). Over 10% of excess deaths were also potentially avoidable for cancers of the kidney (1682, 17%), testis (102, 17%) and uterus (598, 12%), and for Hodgkin disease (189, 12%) and myeloma (986, 11%).

For patients diagnosed during 1990–1994, the largest numbers of avoidable deaths were seen for cancers of the prostate (7335, 20%), breast (6165, 15%), colon (4376, 9%), lung (3735, 2%), stomach (3672, 9%) and kidney (2644, 22%). More than 10% of excess deaths within 5 years of diagnosis were also potentially avoidable for cancers of the uterus (811, 16%) and testis (80, 16%), and for Hodgkin disease (213, 14%).

For patients diagnosed during 1995–1999, most of the avoidable deaths within 5 years arose for cancers of the prostate (5758, 17%), breast (5475, 15%), lung (4923, 3%), colon (4295, 10%), stomach (3137, 9%) and kidney (2686, 21%). At least 10% of excess deaths were also potentially avoidable for cancers of the uterus (524, 11%) and ovary (1801, 10%), and Hodgkin disease (132, 11%).

Compared with the mean survival in 13 European countries that contributed data to the three most recent EUROCARE studies (-2, -3 and -4), avoidable cancer mortality within 5 years of diagnosis in Britain changed from 6614 deaths a year for patients diagnosed during 1985–1989, to 7524 deaths a year for 1990–1994 and 6968 deaths a year for 1995–1999. As a percentage of the overall excess mortality among patients diagnosed with cancers for which there was a survival deficit, avoidable premature mortality was about 6–7% of the total excess mortality within 5 years of diagnosis for all three periods.

Most of the avoidable premature deaths occurred for cancers of the breast, prostate, colon, stomach, lung and kidney. Avoidable mortality has fallen for breast cancer, increased for cancer of the lung, and remained more or less stable for stomach and colon cancers (Figure 2).

For prostate cancer, avoidable mortality rose from the late 1980s to the early 1990s, but fell in the late 1990s. Prostate cancer contributes a large number of avoidable deaths. Compared with the mean survival in Europe, the number of avoidable deaths for all other cancers would change from 6600 (6.3%) to 5757 (5.8%) for patients diagnosed 1985–1989; from 7524 (6.7%) to 6057 (5.8%) for 1990–1994 and from 6968 (6.5%) to 5817 (5.7%) for 1995–1999 (results not shown).

### Highest European survival

**Figure 3:**
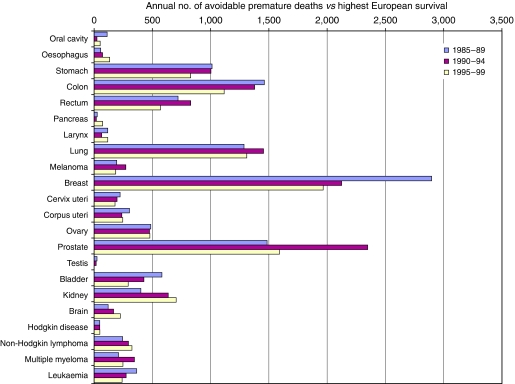
Annual number of deaths within 5 years of diagnosis that would be avoidable among cancer patients in Britain if relative survival were equal to the highest European survival: 22 common cancers, patients diagnosed 1985–1989, 1990–1994, 1995–1999.

Among patients diagnosed during 1985–1989, the highest European survival was higher than in Britain for all the 22 common cancers and for all but two of the 17 less common cancers (oropharynx, choroid). Overall, 64 203 excess deaths (over and above background mortality) within 5 years of diagnosis among patients diagnosed in Great Britain would have been avoided if the highest European survival had been achieved for all cancers diagnosed during this period. This represents some 12 841 avoidable premature cancer deaths per year, or about 12.2% of the excess mortality (Table 2; Figure 3).

For patients diagnosed during 1990–1994, the highest European survival was higher than in Britain for all 39 cancers examined. Overall, 65 534 deaths within 5 years of diagnosis would have been avoided if the highest European survival had been achieved for all cancers. This represents 13 107 premature cancer deaths per year, or about 11.7% of the excess mortality.

For patients diagnosed during 1995–1999, the highest European survival was higher than in Britain for all but one of the 22 common cancers. For testicular cancer, survival in Britain was slightly higher (14 premature deaths avoided, or 3% of the excess mortality). Survival was also slightly higher for two of the 17 less common cancers (choroid, and the vagina and vulva). In all, 57 119 deaths within 5 years of diagnosis would have been avoided if survival in Britain had reached the highest levels observed in the 13 comparator countries. This represents 11 424 premature cancer deaths a year, or some 10.6% of the excess mortality from these cancers.

Compared with the highest survival in Europe, avoidable cancer mortality in Britain fell slightly from about 13 000 deaths a year for patients diagnosed during the 10-year period 1985–1994 to about 11 400 deaths a year for those diagnosed during 1995–1999. As a percentage of the overall excess mortality among cancer patients in Britain, avoidable premature mortality fell from 12.2% to 11.7% to 10.6% over these three periods.

The annual number of avoidable deaths has fallen steadily for cancers of the breast, colon and bladder, and the leukaemias, and to a lesser extent for cancers of the stomach, uterus and cervix. Avoidable premature mortality has risen to some extent for tumours of the oesophagus, pancreas, kidney and brain, and for non-Hodgkin lymphoma.

Compared with the highest survival in Europe, avoidable mortality for prostate cancer rose in the early 1990s, but fell in the late 1990s. Exclusion of prostate cancer would reduce the overall estimate of the annual number of avoidable deaths by 1500–2000. The percentage of avoidable premature mortality among all the other cancers would be 1–2% lower, but the downward trend similar, falling from 11.4% for patients diagnosed during 1985–1989 to 10.3% for 1990–1994 and 9.7% for 1995–1999 (results not shown).

### Sensitivity analyses

After adding 2% to the 5-year relative survival estimates for each country in Great Britain, for each sex and age group, and for each cancer, the estimated number of premature avoidable deaths, compared with the European mean survival, was 5813 per year (5.6%) among patients diagnosed during 1985–1989. The number increased slightly to 6384 deaths per year (5.8%) for patients diagnosed during 1990–1994 and fell to 5600 deaths per year (5.3%) for patients diagnosed during 1995–1999.

After adding 3% to the 5-year relative survival estimates in Britain, similar calculations suggest 5467 avoidable premature deaths per year among patients diagnosed during 1985–1989, representing 5.3% of the total excess mortality among cancer patients diagnosed in that period. The number increased to 5849 (5.3%) for 1990–1994, but fell to 4954 (4.7%) for patients diagnosed during 1995–1999.

## Discussion

The number of deaths among cancer patients within 5 years of diagnosis (‘premature deaths’) that would be avoidable if relative survival in Great Britain were as high as elsewhere in Europe helps to quantify the public health importance of the survival deficit. It tells us how much the excess cancer mortality could be reduced if the mean (or highest) levels of cancer survival in Europe were to be achieved in Britain. For each cancer, the comparison takes account of the level and trends in survival by age and sex in the 13 other European countries we considered, as well as the differences and trends in background mortality between each of those countries and England, Scotland and Wales.

The estimates of avoidable cancer mortality start from the number of patients who were diagnosed each year during the 15 years 1985–1999, and the proportion who survived up to 5 years, after correction for death from other causes. Reducing the number of premature deaths in cancer patients by improving their survival is thus distinct from cancer prevention, which involves long-term reduction in carcinogenic exposures and is evaluated by reduction in the numbers of new cancer patients actually being diagnosed each year. By contrast, reduction of avoidable premature mortality in patients who do develop cancer requires earlier diagnosis and faster access to optimal treatment for all patients.

The *number* of avoidable deaths depends on both the deficit in relative survival and the number of cancer patients diagnosed in Britain. Thus, if the population were one-half of what it is, but incidence and survival remained constant, the number of avoidable deaths would also be reduced by half, but the *percentage* of avoidable deaths would be the same. This percentage refers to the number of deaths in cancer patients in excess of the expected mortality, not to the overall number of deaths in cancer patients. This is because we cannot seek to reduce overall mortality in cancer patients to zero. By contrast, the excess mortality would indeed fall to zero if overall mortality among cancer patients were no different from the level in the general population; in other words, if all cancer patients were cured (see Figure 1). In the medium term, the proportion of deaths that would be avoidable if some target level of survival were achieved in Britain, such as the mean survival in other European countries, provides a useful guide to the public health importance of reducing the survival deficit.

Five-year survival is the most widely used measure for international survival comparisons. We therefore estimated the number of excess deaths within the 5 years after a cancer diagnosis.

We were conservative in defining the mean survival in Europe, by including only those countries that contributed to three consecutive EUROCARE studies (-2, -3 and -4). We were also conservative in defining the highest survival in Europe, taking the average of the three highest estimates by age, sex and cancer after the exclusion of Austria and Switzerland. We only weighted the estimates of the mean and highest survival in Europe by the proportion of cases contributed by a given country to the EUROCARE-4 study, and not by the total number of cases diagnosed in that country.

Comparisons of cancer survival in the EUROCARE study between Britain and the rest of Europe have often been criticised as unfair to Britain, either because cancer registries in other countries supposedly create artefactually high survival (for example by failing to follow up adequately those patients with the lowest survival), or because the registries that contribute to EUROCARE are not ‘representative’ of the whole country. These arguments are weak. All cancer registries contributing to EUROCARE meet the international quality criteria for inclusion in the WHO compendium *Cancer Incidence in Five Continents* (Curado *et al*, 2007). All data sets in the EUROCARE study are subjected to the same quality control procedures. There is little evidence that cancer registration data are of higher quality in Britain than in most other European countries (Capocaccia *et al*, 2003).

For some countries, only a small proportion of the population was covered by registries contributing to EUROCARE, and critics have suggested that survival from these registries may not adequately reflect national survival (Moran *et al*, 2000; Threlfall *et al*, 2001). The mean European survival estimates used here, however, were weighted only by the numbers of cases actually included in the EUROCARE study, not by the size of the national population: in other words, we made no assumption that survival estimates based on sub-national data sets were representative of the whole country.

Restricting the analyses to countries that have contributed to EUROCARE with national cancer registration coverage would not make the comparison more ‘representative’ of Europe. Only Denmark, Finland, Iceland and Slovenia contributed national data to all three EUROCARE studies considered here, while Austria, Estonia, Ireland, Malta, Norway, Slovakia and Sweden either attained national registration coverage since 1985, or did not contribute national data to all three studies. Apart from the United Kingdom, 9 of the 19 countries in EUROCARE-3 and EUROCARE-4 contributed national data. All five Nordic countries contributed national data, and survival in those countries is generally high, but three of the five eastern European countries, where survival is generally low, also contributed national data (Berrino *et al*, 2003). Similarly, though survival in France is generally high, and the French data in EUROCARE have covered about 6% of the national population, a large national study covering 14 *départements* (20% of the population) for patients diagnosed up to 1997 showed very similar survival estimates to those reported in the EUROCARE studies (Grosclaude *et al*, 2007).

For most cancers, even the highest cancer survival estimates for any English region in the late 1980s were below the mean European survival from EUROCARE-2. Even the survival among the most affluent fifth of all English cancer patients, regardless of their region of residence, was lower than the mean European survival. The highest European survival estimates for patients diagnosed with one of eight major cancers (lung, breast, colon, prostate, bladder, stomach, oesophagus and cervix) during the late 1980s were equal to or higher than the highest survival estimates for patients diagnosed in any of the eight NHS Regions of England, or in Scotland, and higher even than in most of the 95 English Health Authorities, where variation was greater (Romanengo *et al*, 2002), or in the 15 Scottish Health Boards during the 1990s (Scottish Cancer Intelligence Unit, 2000). It follows that neither the inclusion of sub-national data for England in EUROCARE-2 and -3 nor the increased coverage of other countries in the later EUROCARE studies is likely to have altered the comparative pattern very much.

Nevertheless, we sought to address these criticisms in several ways.

For trends in the number of avoidable deaths compared with the European average, we examined the impact of further restricting the comparison to Denmark, Finland, Iceland and Sweden (Norway only joined from EUROCARE-3), because their cancer registration systems have national coverage and excellent quality (Parkin *et al*, 1997) and their health systems are broadly similar to that of the United Kingdom. Comparison with the average survival for these four Nordic countries suggested an annual figure of 7465 (7.1%) avoidable cancer deaths in Great Britain within 5 years of diagnosis for patients diagnosed in 1985–1989 (Supplementary Table 1), not massively discrepant with the estimate of 6614 (6.3%) avoidable deaths a year derived by including the data from all the 13 countries considered here.

The estimates of avoidable cancer mortality do depend on the comparability of survival estimates between Britain and the rest of Europe. Avoidable mortality estimates could thus be biased, to the extent that artefacts (e.g. differences between Britain and other countries in diagnostic criteria and investigations, quality of cancer registration data and follow-up, and lead-time bias for cancers subject to mass screening) contribute to the international differences in survival (Berrino *et al*, 1995a, 1997; Coleman, 1999). However, survival in Britain for melanoma of the skin and cancers of the cervix and uterus has often been as high or higher than the European average, and for testicular cancer, as good as the best. Survival for patients aged 15–44 years in Britain is generally as high as the European mean survival, and differences in survival for children are small (Šteliarová-Foucher *et al*, 2004). Since the differences in survival are not systematic, as one might expect if they were entirely attributable to artefacts of data collection or analysis, this provides some evidence against those who simply assert that the EUROCARE findings are incredible (Cookson, 2000; Wilkinson, 2009).

To assess trends in avoidable mortality compared with ‘the best in Europe’, as envisaged by the NHS Cancer Plan (Department of Health, 2000) and the Cancer Reform Strategy (Department of Health, 2007), we were conservative. First, we excluded data from two countries that most often had the highest survival in Europe (Switzerland and Austria). Then, for each cancer, age group and sex, we used a weighted average of the relative survival in the three countries with the highest age-standardised survival for that cancer, to avoid undue influence of the most extreme survival observed in Europe on the estimates of avoidable mortality.

We could also have assumed that both incidence and survival in regional cancer registries contributing to EUROCARE were indeed ‘representative’ of the entire country. That would have produced higher values for the European mean survival, by giving greater weight to data from large countries with low population coverage, such as Germany, which often had higher survival than the European mean survival as we actually defined it. Estimates of avoidable mortality in Britain would have been higher if we had done this.

National ‘representativeness’ should not arise: this is not an international contest between rival national teams. Some registries with national coverage report lower survival than in Britain, and others higher, but it is irrational to dismiss population-based survival estimates for the purpose of comparison solely because they are regional rather than national (Coleman *et al*, 2003). The central issue is whether the estimates of cancer survival in a given country (or a region of a country) that are higher than in Britain can be considered reliable, and whether artefact and/or bias can explain all the differences. If several large populations have attained a level of cancer survival that Britain would wish to emulate, then we need to quantify and explain that difference in terms that help policymakers reduce it. Here, we compared survival in Britain with data from both regional and national registries in 13 European countries. Restricting the comparison to countries with 100% population coverage by registration (or just the Nordic countries) alters the estimate of avoidable mortality, but does not abolish it.

Assuming that survival in Britain was 2–3% higher for each cancer than actually reported in EUROCARE also reduces the estimates of avoidable mortality, but the annual numbers of avoidable deaths are still considerable (Supplementary Tables 2–3). The possible range of avoidable cancer mortality in Britain is highly unlikely to include zero.

Our estimates of the annual number of avoidable cancer deaths are certainly large, whether assessed against the mean or the highest European survival, but they are well below the previous estimates (10 000 and 25 000 deaths a year, respectively), which were acknowledged as crude (Sikora, 1999), and are clearly not plausible.

Prostate cancer contributed a large proportion of the avoidable deaths in each period. If we exclude prostate cancer, the number of avoidable premature deaths a year assessed against the European mean becomes 5800–6000 (5.7–5.8%), compared with 6600–7500 a year using all the data (6.3–6.7%). Assessed against the highest European survival, avoidable premature deaths excluding prostate cancer fell steadily from 11 400 a year (11.4%) to 9800 deaths a year (9.7%). In proportionate terms, this trend is similar to the full estimate: from about 12 800 (12.2%) to 11 400 (10.6%). The rise and fall of avoidable deaths from prostate cancer may reflect the fact that PSA testing became widespread in Britain some 5 years later than in most of the comparator countries (except Denmark), and that the survival deficit between Britain and other European countries for men diagnosed with prostate cancer in the early 1990s had narrowed by the late 1990s.

The estimates of avoidable deaths cover an arbitrary interval of 5 years since diagnosis because 5-year survival data were readily available. If survival differences persist beyond 5 years after diagnosis, the numbers of avoidable deaths will continue to increase with time since diagnosis, and our figures will under-estimate the total avoidable mortality. Conversely, if differences in relative survival decline with time since diagnosis, our figures will over-estimate the total avoidable mortality. Ideally, the number of avoidable deaths would be calculated for each cancer at a time since diagnosis when the survivors have no further excess mortality relative to the general population: the point of ‘cure’ (De Angelis *et al*, 1999). The point of cure varies between cancers, however, and for some cancers it is difficult to identify. Systematic estimates of the proportion of cancer patients cured in Europe have only recently become available: these are limited to patients diagnosed during the period 1995–1999 (Francisci *et al*, 2009).

These analyses suggest that for adult cancer patients diagnosed in Britain during 1995–1999, some 6.5% (6968) of the 105 157 cancer-related deaths that occurred each year within 5 years of diagnosis would have been avoided if cancer survival had been equivalent to the mean European level, and 10.6% (11 424) of the deaths if survival had been equivalent to the highest survival in Europe. Trends in avoidable cancer mortality in Britain by comparison with the European mean survival have been fairly steady over the 15 years 1985–1999, reflecting the fact that survival in Britain has often been rising in parallel with the European mean survival (Verdecchia *et al*, 2009). Trends in avoidable mortality compared with the highest European survival are more favourable, however, reflecting a small but steady improvement in Britain towards the highest levels of cancer survival in Europe.

We have previously estimated that about 2500 deaths a year would have been avoided among adults diagnosed with 1 of 47 cancers in England and Wales during 1986–1990 if socio-economic inequalities in 5-year relative survival had not existed (Coleman *et al*, 2001). There were some differences from the study reported here in method, geographic coverage, calendar period and the cancers included, but those results suggest that socio-economic differences in survival in Britain may represent up to half the avoidable premature mortality compared with the mean survival in Europe.

The results reported here relate to cancer patients in Britain who were all diagnosed before 2000, when the first national cancer plan was introduced. The results will need updating to examine any impact on avoidable mortality of the national cancer plans in England (Department of Health, 2000), Scotland (Scottish Executive Health Department, 2001) and Wales (Cancer Services Co-ordinating Group, 2006).

## Conflict of interest

The authors declare no conflict of interest.

## Figures and Tables

**Table 1:** National population (thousands), coverage (%) by participating registries and contribution to European ‘highest’ survival, by calendar period: countries included in EUROCARE-2, EUROCARE-3 and EUROCARE-4 studies

	**1985–1989 (EUROCARE-2)**			**1990–1994 (EUROCARE-3)**			**1995–1999 (EUROCARE-4)**			
**Country**	**Population**	**Coverage**	**Highest survival^a^**	**Population**	**Coverage**	**Highest survival^a^**	**Population**	**Coverage**	**Highest survival^a^**	**Change in coverage**
Austria	8030	7.8	NA	7930	8.0	NA	7965	100.0	NA	Yes^b^
Denmark	5140	100.0	9	5205	100.0	4	5275	100.0	5	No
**England**	**51 000**	**49.6**	**—**	**49 310**	**62.6**	**—**	**49 331**	**100.0**	**—**	**Yes^b^**
Finland	4986	100.0	12	5023	100.0	12	5132	100.0	14	No
France^c^	56 735	3.0–5.6	15	56 567	2.9–5.6	15	58 738	10.5–14.7	14	Yes^b^
Germany	62 702	1.7	9	82 183	2.8	14	82 012	1.3	17	Yes^b^
Iceland	255	100.0	8	267	100.0	9	271	100.0	7	No
Italy	57 661	9.7	12	56 318	15.3	10	56 876	25.3–27.4	12	Yes^b^
Netherlands^d^	14 951	5.7–20.5	12	15 047	23.7	14	15 567	34.0	16	Yes^b^
Poland	38 119	6.2	2	38 370	6.1	0	38 639	9.0	4	Yes^b^
**Scotland**	**5100**	**100.0**	**—**	**5119**	**100.0**	**—**	**5086**	**100.0**	**—**	**No**
Slovenia	2000	100.0	3	2072	100.0	1	1987	100.0	4	No
Spain^d^	38 959	9.6–12.9	14	38 714	9.6–14.4	17	39 525	12.2–16.3	7	Yes^b^
Sweden	8414	17.5	22	8918	100.0	22	8844	100.0	17	Yes^b^
Switzerland^d^	6712	11.8	NA	6914	11.9	NA	7081	27.1–46.8	NA	Yes^b^
**Wales**		NA	**—**	**2925**	**100.0**	**—**	**2901**	**100.0**	**—**	**Yes^b^**
	360 764			380 882			385 232			

Abbreviation: NA=not applicable.

The following countries did not participate in all three studies, so their data were not included (see text): Belgium (58% coverage of the national population of 10.2 million in EUROCARE-4), Czech Republic (8%,10.2 million), Ireland (100%, 4.1 million), Malta (100%, 0.4 million), Northern Ireland (100%, 1.7 million), Norway (100%, 4.6 million) and Portugal (43%, 10.5 million).

a Number of cancers included in the analyses for which this country contributed one of the three highest age-standardised relative survival estimates in the EUROCARE study (both sexes combined) for this period. Switzerland and Austria are excluded from the ‘highest’ analysis (see text).

b Change between successive EUROCARE studies: *Austria*: Tyrol (E2, E3); national (E4). *England*: 7 registries (E2); 8 registries (E3); national (E4). *France*: 5 registries (E2); 4 registries (E3); 14 registries (E4). *Germany*: Saarland (E2), plus Munich (E3); minus Munich (E4). *Italy*: 9 registries (E2); 13 registries (E3); 21 registries (E4). *Netherlands*: 2 registries (E2); 2 registries (E3); 3 registries (E4). *Poland*: 2 registries (E2, E3); 3 registries (E4). *Spain*: 6 registries (E2); 6 registries (E3); 8 registries (E4). *Sweden*: Southern Region (E2); national (E3, E4). *Switzerland*: 2 registries (E2, E3); 7 registries (E4). *Wales*: not included in E2, national (E3, E4), see text for details.

c These include specialised cancer registries for certain cancers.

d Data for selected cancers in one or more periods.

**Table 2:** Avoidable deaths – number of deaths (and percentage of excess deaths^a^) that would be avoidable in Great Britain within 5 years of diagnosis, based on the mean (or the highest) survival estimates for 13 other countries in Europe: selected cancers, adults (15–99 years) diagnosed during 1985–1989, 1990–1994 and 1995–1999

	**Patients diagnosed in Great Britain during 1985–1989**						**Patients diagnosed in Great Britain during 1990–1994**						**Patients diagnosed in Great Britain during 1995–1999**					
			**Avoidable deaths based on:**						**Avoidable deaths based on:**						**Avoidable deaths based on:**			
			**Mean European survival^b^**		**Highest European survival^b^**				**Mean European survival^b^**		**Highest European survival^b^**				**Mean European survival^b^**		**Highest European survival^b^**	
**Malignancy**	**No. of patients**	**Excess deaths^a^**	**No.**	**%**	**No.**	**%**	**No. of patients**	**Excess deaths^a^**	**No.**	**%**	**No.**	**%**	**No. of patients**	**Excess deaths^a^**	**No.**	**%**	**No.**	**%**
Oral cavity	4107	2164	**43**	2.0	**558**	25.8	4993	2349	**−239**	−10.2	**119**	5.1	5527	2544	**−188**	−7.4	**257**	10.1
Oesophagus	21 959	20 161	**247**	1.2	**282**	1.4	27 409	24 965	**−163**	−0.7	**363**	1.5	29 078	26 418	**246**	0.9	**665**	2.5
Stomach	48 192	42 851	**3483**	8.1	**5059**	11.8	46 755	40 725	**3672**	9.0	**5013**	12.3	41 705	35 632	**3137**	8.8	**4143**	11.6
Colon	74 591	43 995	**4090**	9.3	**7303**	16.6	86 095	47 109	**4376**	9.3	**6889**	14.6	88 908	45 354	**4295**	9.5	**5581**	12.3
Rectum	46 859	28 200	**1691**	6.0	**3600**	12.8	52 605	29 179	**1662**	5.7	**4144**	14.2	56 312	27 866	**1220**	4.4	**2848**	10.2
Pancreas	24 822	24 014	**193**	0.8	**133**	0.6	26 462	25 360	**−76**	−0.3	**95**	0.4	26 002	25 085	**124**	0.5	**363**	1.4
Larynx	8019	2759	**−186**	−6.8	**582**	21.1	8934	3145	**−181**	−5.7	**327**	10.4	9761	3530	**111**	3.2	**585**	16.6
Lung	163 781	153 415	**3548**	2.3	**6432**	4.2	168 693	156 651	**3735**	2.4	**7270**	4.6	156 854	145 571	**4923**	3.4	**6550**	4.5
Melanoma	17 526	3722	**152**	4.1	**969**	26.0	22 059	4247	**354**	8.3	**1358**	32.0	26 469	4205	**220**	5.2	**926**	22.0
Breast	124 499	41 907	**7541**	18.0	**14 483**	34.6	153 354	40 937	**6165**	15.1	**10 619**	25.9	170 651	36 632	**5475**	14.9	**9831**	26.8
Cervix uteri	20 656	7748	**302**	3.9	**1116**	14.4	18 170	6611	**204**	3.1	**983**	14.9	14 253	5293	**462**	8.7	**899**	17.0
Corpus uteri	17 562	4967	**598**	12.0	**1518**	30.6	19 170	5182	**811**	15.7	**1191**	23.0	20 711	4923	**524**	10.6	**1230**	25.0
Ovary	22 861	16 102	**1064**	6.6	**2431**	15.1	25 241	17 567	**1670**	9.5	**2377**	13.5	26 261	17 754	**1801**	10.1	**2394**	13.5
Prostate	54 318	30 015	**4285**	14.3	**7422**	24.7	77 728	36 221	**7335**	20.3	**11 739**	32.4	103 045	33 219	**5758**	17.3	**7958**	24.0
Testis	6098	600	**102**	17.1	**123**	20.5	7311	499	**80**	16.0	**89**	17.8	8676	392	**−39**	−10.0	**−14**	−3.4
Bladder	52 697	19 429	**−232**	−1.2	**2910**	15.0	59 173	19 650	**1048**	5.3	**2136**	10.9	55 925	20 320	**752**	3.7	**1462**	7.2
Kidney	15 922	9887	**1682**	17.0	**2007**	20.3	20 445	12 093	**2644**	21.9	**3183**	26.3	23 212	12 697	**2686**	21.2	**3521**	27.7
Brain	12 714	10 778	**367**	3.4	**602**	5.6	15 331	12 872	**262**	2.0	**836**	6.5	16 224	13 872	**295**	2.1	**1127**	8.1
Hodgkin disease	5830	1628	**189**	11.6	**244**	15.0	5987	1482	**214**	14.4	**245**	16.5	6122	1218	**132**	10.8	**247**	20.3
Non-Hodgkin lymphoma	25 195	14 130	**603**	4.3	**1224**	8.7	32 180	16 967	**1311**	7.7	**1475**	8.7	36 941	18 014	**632**	3.5	**1625**	9.0
Multiple myeloma	11 497	9200	**986**	10.7	**1049**	11.4	13 193	10 147	**847**	8.4	**1731**	17.1	14 507	10 581	**703**	6.6	**1238**	11.7
Leukaemia	16 352	11 832	**535**	4.5	**1828**	15.4	22 894	14 797	**224**	1.5	**1376**	9.3	25 703	15 018	**191**	1.3	**1201**	8.0
*Other cancers* c																		
GB survival higher^d^	7211	2592	**−150**	−5.8	**−108**	−4.2	15 214	6459	**−244**	−3.8	**0**	NA	22 406	11 215	**−623**	−5.6	**−107**	−1.0
GB survival lower^d^	36 283	24 174	**1370**	5.7	**2328**	9.6	37 122	25 504	**1006**	3.9	**1976**	7.7	35 533	22 581	**1154**	5.1	**2468**	10.9
*All cancers (over 5-year period)*																		
GB survival higher^d^	67 927	24 780	**−568**	−0.1	**−108**	0.0	83 012	62 278	**−903**	−0.2	**0**	0.0	36 609	14 151	**−850**	−0.2	**−121**	0.0
GB survival lower^d^	771 624	501 490	**33 071**	6.3	**64 203**	12.2	883 506	498 440	**37 620**	6.7	**65 534**	11.7	984 177	525 783	**34 841**	6.5	**57 119**	10.6
Total	839 551	526 270					966 518	560 718					1 020 786	539 934				
																		
*All cancers (annual avoidable deaths)*																		
GB survival higher^d^			**−114**		**−22**				**−181**		**0**				**−170**		**−24**	
GB survival lower^d^		**100 298**	**6614**		**12 841**			**99 688**	**7524**		**13 107**			**105 157**	**6968**		**11 424**	

a Difference between the number of deaths observed among cancer patients within 5 years of diagnosis, and the number expected from background mortality by age and sex in Britain (see text).

b Based on relative survival estimates from EUROCARE-2 study for 1985–89 patients, EUROCARE-3 study for 1990–94 patients and EUROCARE-4 study for 1995–99 patients (see text).

c Sum for 17 other cancers, analysed separately (data not shown).

d Numbers of avoidable deaths summed separately according to whether survival in Great Britain was higher or lower than the mean (or highest) European survival estimate.

## Appendix

For each cancer, we first calculate a weight for each age, sex and country, *w*_*ijm*_, as the number of patients, *n*, of that age and sex in that country, divided by the total number of cases (all countries):

where *i* indexes age, *j* sex and *m* country.

If there were no cases (and thus no survival estimate) in a given age–sex group, we assumed that there was one case, for the purposes of calculation of the weight, and that survival was equal to that in the adjacent age group. This enabled calculation of a full set of weights, but gave virtually zero weight to the imputed survival for that age–sex group in the weighted European average. This approach ensured that for a given cancer, the sum of the weights in a given calendar period was always unity. The weights from the EUROCARE-4 data were applied to the data for each calendar period. For bone cancer, however, the youngest age group was 20–44 years for the first two studies, but 15–44 years in EUROCARE-4, so the EUROCARE-3 weights were applied to both EUROCARE-2 and -3 data.

For a given cancer, the European mean (or highest) relative survival, RS_E_, in each age and sex group, is then the weighted sum across countries of the age-sex-specific relative survival estimates:

where *m*=13 countries for the mean European survival and 3 for the highest European survival.

Then, for each cancer and each calendar period of diagnosis (1985–1989, 1990–1994, 1995–1999) and each country (England, Scotland, Wales), the number of avoidable deaths within 5 years of diagnosis in each age group and sex is calculated as the product of the number of cases, the expected survival and the difference between the mean (or highest) 5-year relative survival for each age and sex group in Europe and the corresponding relative survival in England, Scotland, Wales. This product is summed over age, sex and country in Great Britain for each 5-year period, and divided by 5 to present an annual number of avoidable deaths:

where, for each age group *i* and each sex *j*:

*N*_C_ is the number of cases diagnosed in country C (England, Scotland or Wales) during the 5-year periods 1985–1989, 1990–1994 or 1995–1999. For 1985–1989, the number of cases for Wales was assumed to be 6% of the number for England, and survival in Wales was assumed to be the same as in England; *ES*_*C*_ is the expected survival for country *C* (England, Scotland or Wales); *RS*_*E*_ is the *mean* or *highest* relative survival, as defined (see text), in EUROCARE-2 (1985–1989), EUROCARE-3 (1990–1994) or EUROCARE-4 (1995–1999); *RS*_*C*_ is the relative survival for country *C* (England, Scotland or Wales).
